# MorCVD: A Unified Database for Host-Pathogen Protein-Protein Interactions of Cardiovascular Diseases Related to Microbes

**DOI:** 10.1038/s41598-019-40704-5

**Published:** 2019-03-11

**Authors:** Nirupma Singh, Venugopal Bhatia, Shubham Singh, Sonika Bhatnagar

**Affiliations:** 1 Computational and Structural Biology Laboratory, Division of Biological Sciences and Engineering, Netaji Subhas University of Technology, Dwarka, New Delhi 110078 India; 2 Division of Computer Engineering, Netaji Subhas University of Technology, Dwarka, New Delhi 110078 India

## Abstract

Microbe induced cardiovascular diseases (CVDs) are less studied at present. Host-pathogen interactions (HPIs) between human proteins and microbial proteins associated with CVD can be found dispersed in existing molecular interaction databases. MorCVD database is a curated resource that combines 23,377 protein interactions between human host and 432 unique pathogens involved in CVDs in a single intuitive web application. It covers endocarditis, myocarditis, pericarditis and 16 other microbe induced CVDs. The HPI information has been compiled, curated, and presented in a freely accessible web interface (http://morcvd.sblab-nsit.net/About). Apart from organization, enrichment of the HPI data was done by adding hyperlinked protein ID, PubMed, gene ontology records. For each protein in the database, drug target and interactors (same as well as different species) information has been provided. The database can be searched by disease, protein ID, pathogen name or interaction detection method. Interactions detected by more than one method can also be listed. The information can be presented in tabular form or downloaded. A comprehensive help file has been developed to explain the various options available. Hence, MorCVD acts as a unified resource for retrieval of HPI data for researchers in CVD and microbiology.

## Introduction

Cardiovascular diseases (CVDs) are amongst the most common cause of mortality and account for high morbidity across the globe^1,2^. Some of the major cardiovascular diseases include cardiac hypertrophy, rheumatic heart disease, ischemic heart disease, coronary artery disease, peripheral artery disease, and cerebrovascular disease^3^. In the past few years, the paradigm that microorganisms play an important role in the initiation and progression of CVDs has emerged. This paradigm has been supported by multiple epidemiological studies that have established positive associations between the risk of cardiovascular disease and markers of infection. Evidence implicating the infection by microbes in CVD includes the identification of viruses and bacteria in atherosclerotic plaques^4^, sero-epidemiological data^5^, and a strong association between specific infections such as Cytomegalovirus with transplant atherosclerosis^6,7^.

Common cardiovascular diseases caused by infection with microorganisms are endocarditis, pericarditis and myocarditis^8^. Infectious organisms or their structural components show the ability to induce proatherogenic and prothrombotic responses in cells relevant to atherogenesis (smooth muscle cells, monocyte-macrophages, T-cells, and endothelial cells)^9^. Microbial species that are found to be present in CVD affected patient samples include *Chlamydia pneumoniae*, *Porphyromonas gingivalis*, *Helicobacter pylori*, Influenza virus, Hepatitis C virus, Cytomegalovirus, Human Immunodeficiency Virus, Coxsackie Virus, and *Staphylococcus* species^10^. The mechanism of interaction of these microbial species with the human system at the molecular level and their involvement in the initiation, progression and severity of CVDs is yet to be elucidated.

Currently available CVD related databases like CardioGenBase database^11^, CADgene database^12^ provide molecular and protein-protein interactions (PPIs) information but do not cover any HPI information of CVDs caused by microorganisms. Several databases list HPI data at the level of interacting proteins e.g. Reactome^13^, HMDAD^14^, PHI-base^15^, VirusMentha^16^, OrthoHPI^17^, VirusMINT^18^, EHFPI^19^, MatrixDB^20^, BioGrid^21^, HPIDb^22^, MINT^23^, IMEx^24^, IntAct^25^, UniProt^26^, MPIDB^27^, VirHostNet^28^, I2D^29^, InnateDB^30^, DIP^31^, Mentha^32^ and PHISTO^33^. Of these, only BioGrid, HPIDb, MINT, IntAct, UniProt, MPIDB, VirHostNet, I2D, MatrixDB, InnateDB and DIP contain limited and scattered information of HPIs leading to CVDs.

At present, independently collected host pathogen protein interaction data in microbe induced CVD is housed in various databases. This poses a big problem since the interactions across all these databases are repeated, scattered or highly fragmented. At present, no database is available that comprehensively lists all the unique protein interactions between host and pathogen in CVDs in a standard, enriched format. A researcher requiring such data has to first take the pains of aggregating the data from various databases available online. The data then has to be filtered, cleaned, processed and verified before finally being used. Therefore, we have developed a new database named MorCVD solely dedicated to the information comprising the interactions between proteins of human and microbial species leading to different types of CVD.

The keywords pertaining to microbe induced CVDs were finalized initially and a list of genes associated with those keywords data was collected from relevant databases. The HPIs corresponding to the genes were mined separately from twelve different databases, cleaned and enriched. For each interaction, gene ontologies, drug target data and interactors are available in MorCVD. The protein, literature and ontology records have been integrated through hyperlinks. The MorCVD MySQL database has a web interface developed using HTML, asp.net framework, CSS, JQuery, JavaScript and Microsoft Visual Studio. Several search options were developed to allow query of the database namely “Disease”, “Pathogen-Specific Interactions”, “Protein-Specific Interactions”, “Gene Ontologies”, “Interaction Detection Methods” and Interactors and Drug Targets”. MorCVD database is freely accessible at http://morcvd.sblab-nsit.net/About and will act as a unique resource for researchers in the field of microbiology and CVD.

## Results and Discussion

The MorCVD database provides comprehensive information on human and pathogen proteins involved in the interactions leading to CVDs. The web application is compatible with multiple browsers like Chrome, Internet Explorer, Firefox, Safari and Microsoft Edge.

After processing the data collected from different databases, 23,377 unique HPIs were obtained. The associated data fields of the database were pathogen name, taxon category, taxonomy id, UniProt accession number, UniProt entry name, gene symbol, gene id, gene ontology, secondary interactors, drug target information of host and pathogen proteins, types of interaction, interaction detection methods, and confidence score of the interaction along with relevant PubMed IDs. The data fields like protein IDs, Pubmed IDs and gene ontology records were associated with corresponding clickable hyperlinks.

**Figure 1:**
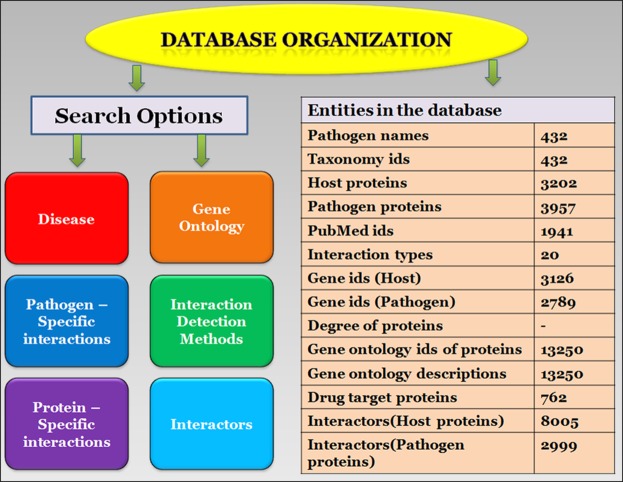
A schematic of database organization. Search options of the database are shown on the left and count of occurrence of each entity in the data is listed on the right.

**Figure 2:**
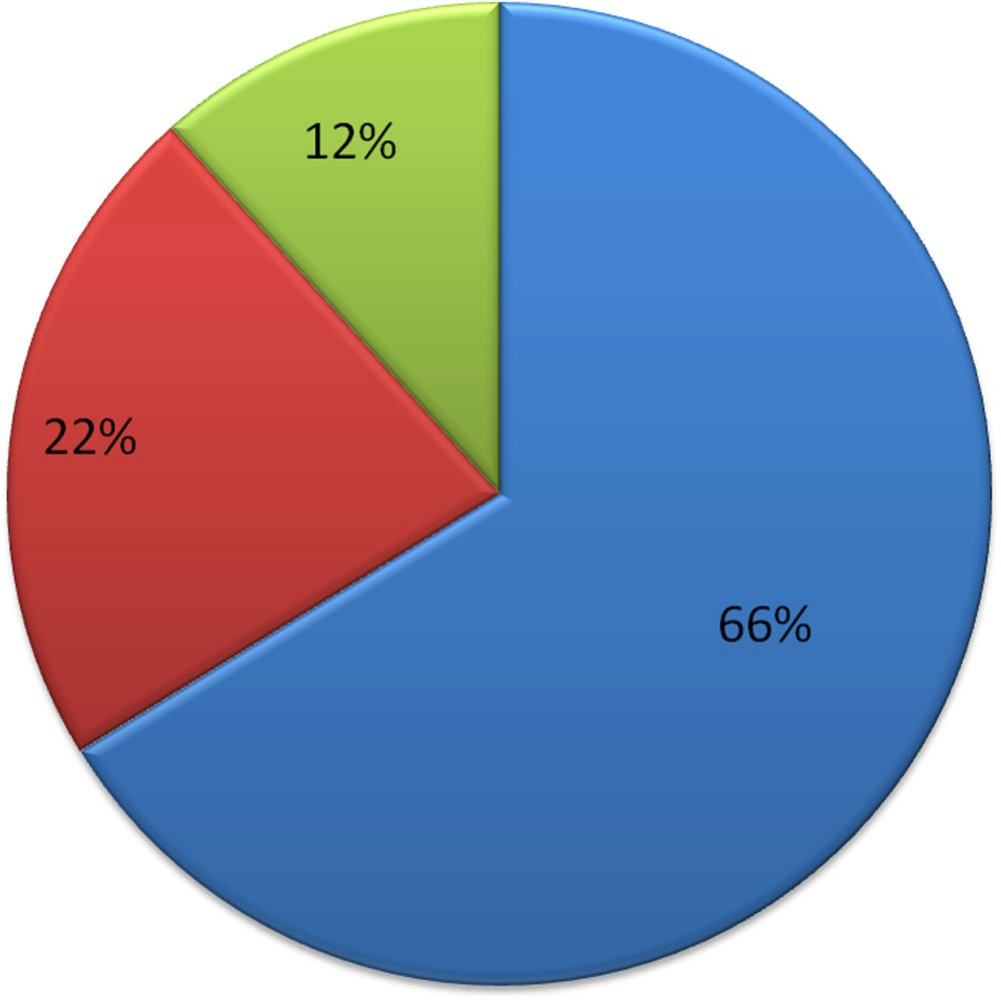
Pie chart showing the frequency of different types of pathogens in the database. Red denotes bacterial species; blue represents virus and green shows fungi as well as other pathogen species.

The confidence scores are assigned to host pathogen interactions to have a metric for gauging the likelihood of an interaction being biologically significant. These scores combine a variety of scoring schemes based on the number of publications supporting the interaction, number of detection methods, interaction types, shared gene ontologies etc. The confidence score for each HPI in MorCVD has been taken from the respective database from which the interaction data was originally obtained. The database was organized according to the broad schema shown in Fig. 1. The frequency of different types of pathogen species is shown in the form of a pie chart in Fig. 2.

**Figure 3:**
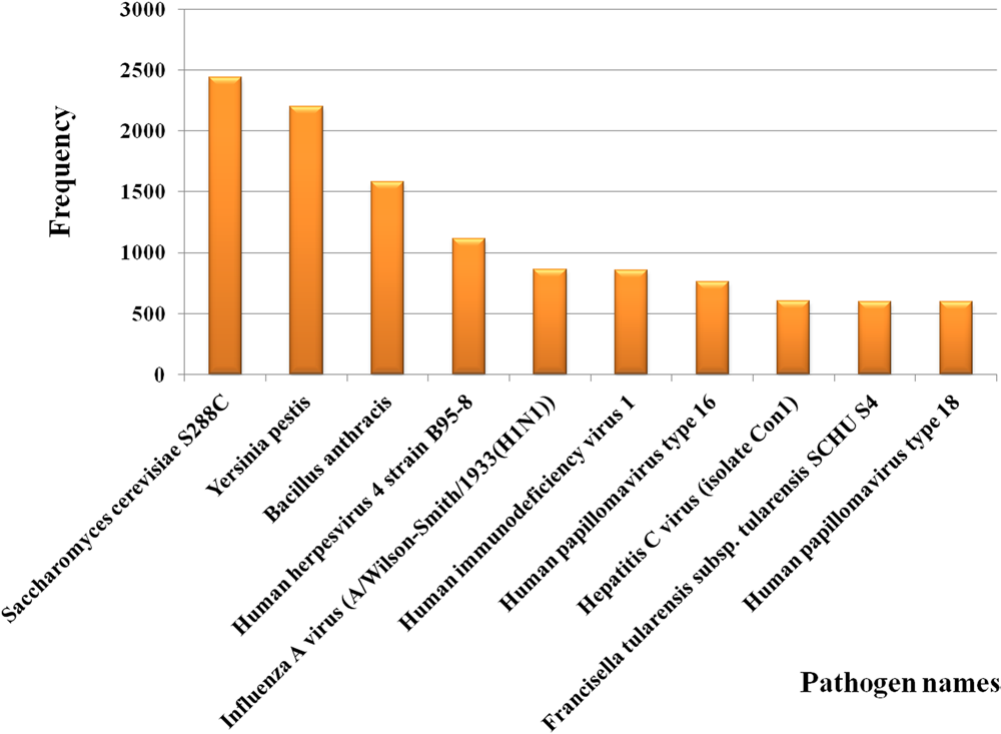
Frequency distribution of pathogens. The bar graph shows the number of top 10 pathogens in the database.

**Figure 4:**
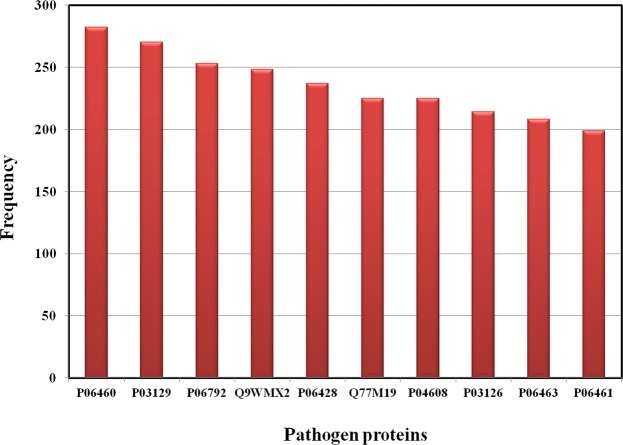
Frequency distribution of pathogen proteins. The bar graph shows the number of top 10 pathogen proteins in the database. The description of UniProt accession number for pathogen proteins is as follows: P06460 - Probable protein E5A(Human papillomavirus type 6b), P03129 - Protein E7 (Human papillomavirus type 16), P06792 - Probable protein E5 (Human papillomavirus type 18), Q9WMX2 - Genome polyprotein (Hepatitis C virus genotype 1b (isolate Con1) (HCV)), P06428 - Protein E6 (Human papillomavirus type 8), Q77M19 - V protein (Measles virus (strain Edmonston-Schwarz vaccine) (MeV)), P04608 - Protein Tat (Human immunodeficiency virus type 1 group M subtype B (isolate HXB2) (HIV-1)), P03126 - Protein E6 (Human papillomavirus type 16), P06463 - Protein E6 (Human papillomavirus type 18), P06461 - Probable protein E5B (Human papillomavirus type 6b).

**Figure 5:**
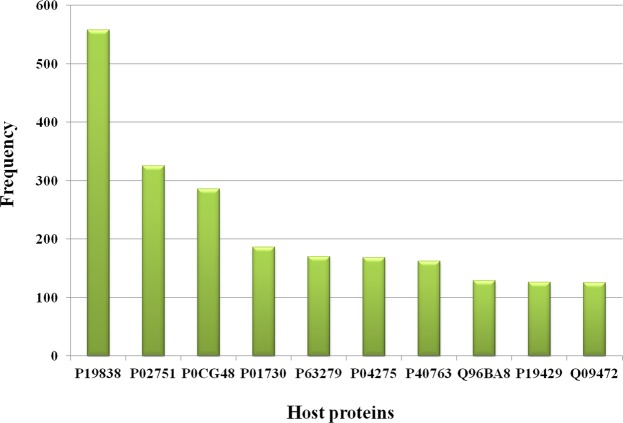
Frequency distribution of host proteins. The bar graph shows the number of top 10 host proteins in the database. The description of UniProt accession number for human host proteins is as follows: P19838 - Nuclear factor NF-kappa-B p105 subunit; P02751 - Fibronectin; P0CG48 - Polyubiquitin-C; P01730 - T-cell surface glycoprotein CD4; P63279 - SUMO-conjugating enzyme UBC9; P04275 - von Willebrand factor; P40763 - Signal transducer and activator of transcription 3; Q96BA8 - Cyclic AMP-responsive element-binding protein 3-like protein 1; P19429 - Troponin I, cardiac muscle; Q09472 - Histone acetyltransferase p300.

The top 10 most frequently occurring pathogens, pathogen proteins and host proteins in the database along with their frequencies are shown in Figs 3, 4 and 5 respectively. The number of interactions that were found for each of the disease term searched is shown in Table 1. Among all the interactions the disease ‘viral myocarditis’ was found to have the maximum number of interactions in the database. The number of HPIs for each type of pathogen involved in microbial CVDs (Table 2) and their frequency by class (Fig. 2) showed that till date virus-host interactions have been most widely studied in CVDs. Among viral species, predominantly occurring viruses were Human herpesvirus 4 strain B95-8,Influenza A virus (A/Wilson-Smith/1933(H1N1)), Human immunodeficiency virus 1, Human papillomavirus type 16 and Hepatitis C virus (isolate Con1). In case of bacterial species, the predominant microorganisms were *Saccharomyces cerevisiae* S288C, *Yersinia pestis*, *Bacillus Anthracis* and *Francisella tularensis* subsp. *tularensis* SCHU S4.

**Table 1:** Number of interactions for each disease term.

S. No.	Disease name	Number of interactions
1.	Viral Myocarditis	8002
2.	Dilated Cardiomyopathy	6155
3.	Endocarditis	3541
4.	Cardiovascular Infections	2786
5.	Hypereosinophilic Syndrome	749
6.	Pericarditis	675
7.	Myocarditis	495
8.	Bacterial Endocarditis	345
9.	Infective Endocarditis	273
10.	Peripartum Cardiomyopathy	100
11.	Native Valve Endocarditis	78
12.	Subacute Bacterial Endocarditis	53
13.	Acute Myocarditis	42
14.	*Staphylococcus aureus* Endocarditis	34
15.	Viral Cardiomyopathy	17
16.	Q-fever Endocarditis	16
17.	Chronic Myocarditis	8
18.	Endocarditis of mitral valve	5
19.	Camptodactyly-Arthropathy-Coxa Vara-Pericarditis Syndrome	3

**Table 2:** Types of pathogen involved with number of interactions.

S.No.	Pathogen	Number
1.	Virus	15514
2.	Bacteria	5184
3.	Fungi	2668
4.	Protozoa	24
5.	Amoebozoa	9
6.	Archaea	2

**Figure 6:**
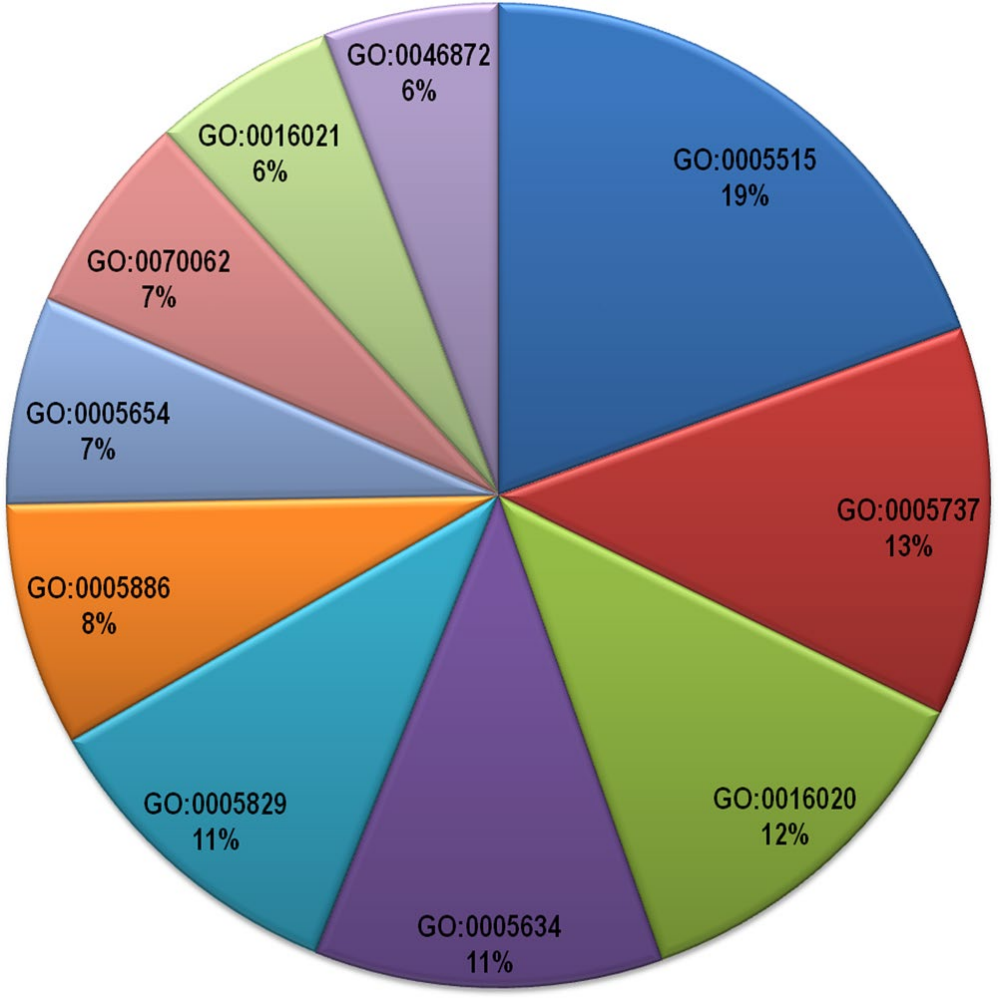
Pie Chart showing frequency of top 10 most prominent gene ontologies of host proteins in the database. The gene ontology description is as follows: GO:0005515 -protein binding, GO:0005737 - cytoplasm, GO:0016020 - membrane, GO:0005634 - nucleus, GO:0005829 - cytosol, GO:0005886 - plasma membrane, GO:0005654 - nucleoplasm, GO:0070062 - extracellular exosome, GO:0016021 - integral component of membrane, GO:0046872 - metal ion binding.

**Figure 7:**
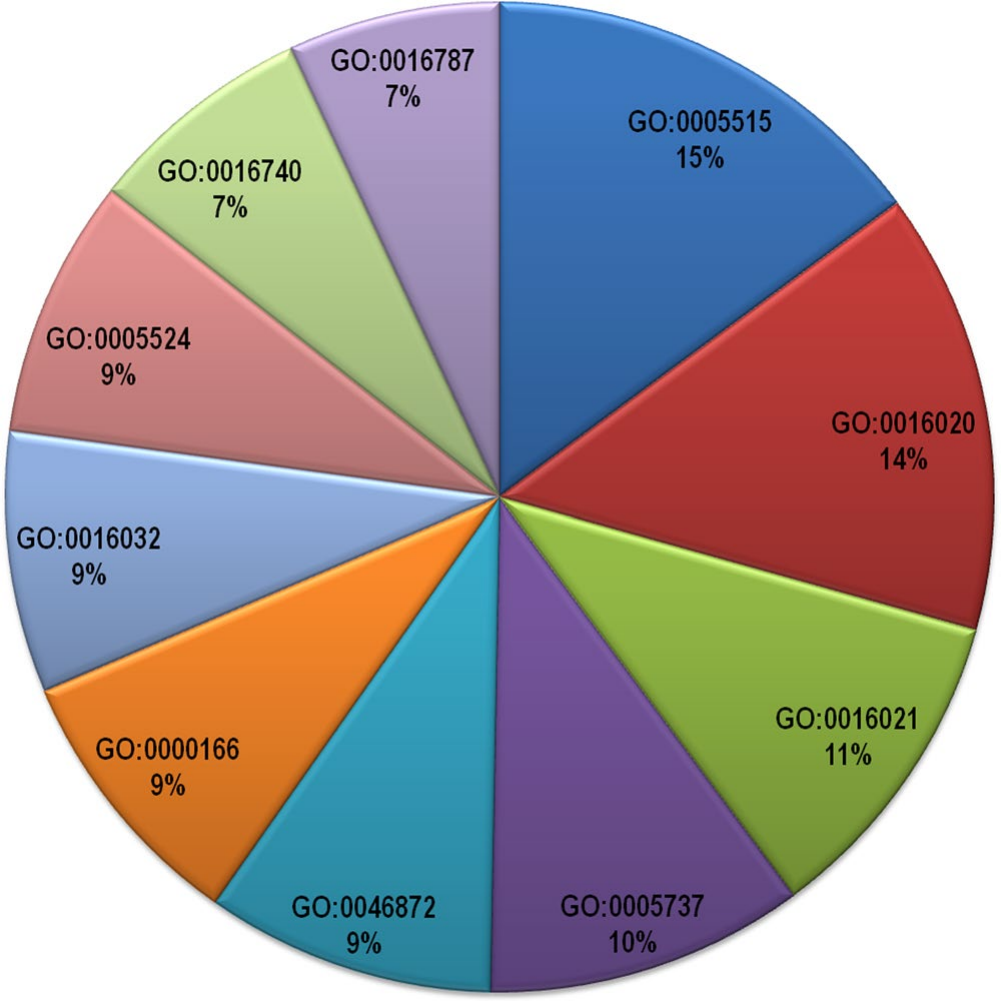
Pie Chart showing frequency of top 10 most prominent gene ontologies of pathogen proteins in the database. The gene ontology description is as follows: GO: 0005515 - protein binding, GO:0016020 - membrane, GO:0016021 - integral component of membrane, GO:0005737 - cytoplasm, GO:0046872 - metal ion binding, GO:0000166 -nucleotide binding, GO:0016032 - viral process, GO:0005524 - ATP binding, GO:0016740 - transferase activity, GO:0016787 - hydrolase activity.

The enrichment of initial entities was further done for better analysis of the data. The parameters considered for the enrichment were gene ontologies, drug target information and interactors (same and different species) of host and pathogen proteins. Gene ontology is an important parameter to consider for enrichment analysis because it provides the information about biological process, molecular function and cellular component of the proteins^34^. By considering these three aspects of gene ontologies we get an idea about overrepresented or underrepresented functions and processes of proteins in our data as well as the location of proteins in a particular disease condition. The pathogen first has to make contact with the host via either extracellular secreted or membrane host proteins and further PPIs take place in the cytoplasmic environment. Therefore, the proteins were found equally enriched in all the cellular compartments like cytoplasm (GO:0005737), membrane (GO:0016020) and integral component of membrane (GO:0016021) and were further scanned for enriched biological process and molecular function. The gene ontology molecular functions like protein binding(GO:0005515) and metal ion binding (GO:0046872)were found to be most enriched in the data both in case of human host as well as the pathogen. Some other gene ontologies those were found to be predominantly occurring exclusively in case of pathogen proteins were molecular functions like nucleotide binding(GO:0000166) and ATP binding(GO:0005524).The predominantly occurring gene ontology biological process in case of host proteins was regulation of transcription (GO:0006355) while viral process (GO:0016032) in case of pathogen proteins. The top ten gene ontologies for host and pathogen proteins are listed in Table 3 and their frequencies are represented in the form of a pie chart in Figs 6 and 7 respectively.

**Table 3:** Predominant occurring gene ontology ids (top 10).

S. No.	Gene Ontology (human)	Gene Ontology (pathogen)
1.	protein binding (GO:0005515)	protein binding (GO:0005515)
2.	cytoplasm (GO:0005737)	membrane (GO:0016020)
3.	membrane (GO:0016020)	integral component of membrane (GO:0016021)
4.	nucleus (GO:0005634)	cytoplasm (GO:0005737)
5.	cytosol (GO:0005829)	metal ion binding (GO:0046872)
6.	plasma membrane (GO:0005886)	nucleotide binding (GO:0000166)
7.	nucleoplasm (GO:0005654)	viral process (GO:0016032)
8.	extracellular exosome (GO:0070062)	ATP binding (GO:0005524)
9.	integral component of membrane (GO:0016021)	transferase activity (GO:0016740)
10.	metal ion binding (GO:0046872)	hydrolase activity (GO:0016787)

After gene ontology enrichment analysis, drug target analysis was done. A search for those proteins in the data identified 702 human proteins and 60 pathogen proteins as previously known drug targets. Drug target analysis was done to look for gene ontology pattern of drug targets and their interactions in the data. The frequently occurring gene ontologies for both human and pathogen drug target proteins were found to be molecular functions like protein binding (GO:0005515) and metal ion binding (GO:0046872). The prominent gene ontology biological processes in case of pathogen drug targets were the viral process (GO:0016032) and proteolysis (GO:0006508). The molecular functions more prominent exclusively in case of pathogen drug target proteins were hydrolase activity (GO:0016787) and transferase activity (GO:0016740).

The quantitative analysis done for the number of interactions between host and pathogen proteins provided a list of high degree proteins. It is important to consider the highly interacting host proteins because they represent the most influential entities in the data and these are the proteins approached most by the pathogen proteins. We found 30 host proteins and 69 pathogen proteins in our data that were high degree. The top 5 high degree host and pathogen proteins in the data are listed in Table 4. Amongst the host drug target proteins, the ones having the highest degree were Nuclear factor NF-kappa-B p105 subunit protein, Fibronectin, Polyubiquitin-C, T-cell surface glycoprotein CD4 and von Willebrand factor while in case of pathogen they were Genome polyprotein (Hepatitis C virus genotype 1b (isolate Con1) (HCV)), Genome polyprotein (Hepatitis C virus genotype 1a (isolate H) (HCV)) and Pol polyprotein (Human immunodeficiency virus 1).

**Table 4:** High degree (highly interacting) proteins of data (top 5).

S. No.	Host proteins	Pathogen proteins
1.	Nuclear factor NF-kappa-B p105 subunit	Probable protein E5A (Human papillomavirus type 6b)
2.	Fibronectin	Protein E7 (Human papillomavirus type 16)
3.	Polyubiquitin-C	Probable protein E5 (Human papillomavirus type 18)
4.	T-cell surface glycoprotein CD4	Genome polyprotein (Hepatitis C virus genotype 1b (isolate Con1) (HCV))
5.	SUMO-conjugating enzyme UBC9	Protein E6 (Human papillomavirus type 8)

In the last part of the analysis same and different species interactors for both host and pathogen proteins were added. It is important to study about all types of interactors because they together regulate a variety of cellular functions, including cell cycle progression, signal transduction, and metabolic pathways inside the body^35^. The proteins having a very large number of interactors are known to be essential as they play a key role in the metabolism of the body and can disturb the functioning of the body if troubled. In our data, the human and pathogen proteins with maximum number of interactors are listed in Table 5.

**Table 5:** Proteins with maximum number of interactors (same and different species) (top 5).

S. No.	Host proteins	Pathogen proteins
1.	Homeobox protein MOX-2	Ribosome-associated molecular chaperone SSB1 (*Saccharomyces cerevisiae* (strain ATCC 204508/S288c) (Baker’s yeast))
2.	Microtubule-associated tumor suppressor candidate 2	Alpha N-terminal protein methyltransferase 1 (*Saccharomyces cerevisiae* (strain ATCC 204508/S288c) (Baker’s yeast))
3.	MyoD family inhibitor	Translation initiation factor eIF-2B subunit beta (*Saccharomyces cerevisiae* (strain ATCC 204508/S288c) (Baker’s yeast))
4.	Proto-oncogene c-Rel	ATP-dependent molecular chaperone HSC82 (*Saccharomyces cerevisiae* (strain ATCC 204508/S288c) (Baker’s yeast))
5.	Cellular tumor antigen p53	Heat shock protein SSA1 (*Saccharomyces cerevisiae* (strain ATCC 204508/S288c) (Baker’s yeast))

After all the MySQL work done for database construction and analysis, a web interface named MorCVD database was developed for this data. It comprises several search options that fulfill the demand for extraction of HPI data and its enrichment parameters. They are as follows:

- **Disease**
  This option can be used to list all the HPIs related to a particular disease term. It requires the selection of a specific term from the drop-down menu of 19 disease terms displayed on the webpage. The user can choose any one of the disease terms to retrieve the desired data. The search result leads to the record of alphabetically ordered pathogen names, pathogen taxonomy identifiers, UniProt accession numbers, gene symbols and UniProt entry names, of host and pathogen proteins, a sortable confidence score, source database for the interaction and respective PubMed reference of the HPI. For every disease, results pertaining to specific host or pathogen protein can be found using the “Search by protein IDs” tab present on the result page. The data download option makes it convenient for the user to explore large number of results simultaneously.
- **Pathogen-specific interactions**
  This option allows the user to look for HPIs with respect to a particular pathogen query. It comprises a pathogen tab that lists all the pathogen names in a drop-down menu. When a pathogen is selected all the HPIs related to that particular pathogen are listed. This could help in determining the list of proteins interacting in the data for a particular pathogen.
- **Protein-specific interactions**
  This option allows the querying of the database by a particular protein. After the selection of host (i.e. either human host or pathogen-host), the option requires the input of respective protein’s UniProt accession number. It then lists all the interactions related to that particular protein along with their gene identifiers, degrees of the listed proteins and the corresponding disease. In case of selecting pathogen as host, the user further needs to select a pathogen from the drop-down menu and then provide the UniProt accession number of protein for that particular pathogen.
- **Gene Ontologies**
  This option can be used to provide the gene ontology information of host and pathogen proteins present in the data. The user needs to provide UniProt accession numbers of either human or pathogen proteins in order to list the corresponding gene ontology identifiers with their class, description and quantitative value (on the basis of its occurrence in the data) of each gene ontology id.
- **Interaction – detection methods**
  This option lists the HPIs in the data by a particular interaction detection method. After selection of an interaction detection method from the drop-down menu, it lists all the HPIs that were identified by that particular interaction detection method along with interaction type between them. With the help of this option, we also get information about the type of interactions fetched out from a particular interaction detection method.
  A separate page provides the option to search the interactions detected by multiple methods. The user has to select the number of methods from the drop down menu to obtain the interactions along with the respective interaction detection methods.
- **Interactors and Drug targets**

This option provides the list of interactors of the human and pathogen proteins. The user can list the interactors of a particular protein by choosing a host and then providing the UniProt accession number of either the human or pathogen protein as input. This option also provides the drug target information of the queried protein, whether it is a drug target or not. The user can also look for common interactors between two proteins.

Hence, MorCVD is a database constructed to provide a comprehensive view of molecular information of HPIs leading to CVDs. MorCVD encompasses a broader spectrum of data and provides us a unique resource that collates all the HPIs involved in CVDs by integrating the information from biological databases present in a scattered manner. By providing effective search and browsing features, it operates as a flexible and user-friendly platform for the molecular study of microbial CVDs. It contains the gene ontology parameters, drug target information and interactors within the same species as well as different species of host and pathogen proteins. Comprehensive documentation of the database is available to the users through the Documentation option (Supplementary File 1). A link to a page containing a brief description of all the 116 detection methods for determining the interactions has been included in the Documentation (Supplementary Table S1 in Supplementary File 1).

MorCVD database gives a collective idea about biochemical and physiological properties of proteins. Analysis of the data provided the information that viruses are more likely to be involved in case of microbial CVDs as compared to other pathogen species as determined by the frequency of viruses in the HPI data. MorCVD lists enriched HPI data relevant to CVDs and is sufficient to show that most of the interactions have been determined for virus proteins and many more bacterial HPIs need to be determined. It also provides the information about already known drug target proteins present in our data. More number of host proteins were identified as drug targets in the data as compared to pathogen proteins. Thus, MorCVD is a unified database which provides the information about HPIs involved in microbial CVDs, their functional features, and some biological properties.

### Methods

The building of the database included the following steps:

1. *Database mining and curation*
  The information for the database was collected by defining a list of search terms through a literature survey. The infection of microorganisms in the heart is classified mainly into three types of cardiovascular inflammatory disorders namely ‘endocarditis’, ‘myocarditis’ and ‘pericarditis^8,36^. Consecutively, some other terms were also found from DisGeNet database^37^ associated with former three main disease terms and were cross-checked from the literature. Those terms were ‘dilated cardiomyopathy’, ‘viral cardiomyopathy’, ‘viral myocarditis’, ‘acute myocarditis’, ‘chronic myocarditis’, ‘peripartum cardiomyopathy’, ‘Camptodactyly-Arthropathy-Coxa Vara-Pericarditis Syndrome’, ‘hypereosinophilic syndrome’, ‘bacterial endocarditis’, ‘infective endocarditis’, ‘Q – fever endocarditis’, ‘subacute bacterial endocarditis’, ‘*Staphylococcus aureus* endocarditis’, ‘native valve endocarditis’, ‘endocarditis of mitral valve’ and ‘cardiovascular infections’. After defining the list of search terms, genes related to them were extracted from the databases, namely OpenTargets^38^ and DisGeNet.
  The combined gene list of both the databases after removing duplicates was fed into the following databases i.e. BioGrid, HPIDb, MINT, IntAct, UniProt, MPIDB, VirHostNet, I2D, MatrixDB, InnateDB and DIP in order to extract the relevant HPI information. HPI data collected from these resources included the corresponding UniProt accession numbers, UniProt entry names and gene symbols for interacting proteins of human host and pathogen, interaction detection method, confidence score of the interaction, interaction type between the two proteins, pathogen names for pathogen proteins with their taxon id and taxon category and PubMed id reference of the HPI. There were also some HPI databases that did not provide any relevant data for our objective, namely Reactome, HMDAD, PHI-base, VirusMentha, OrthoHPI, VirusMINT and EHFPI.
2. *Data processing*
  The raw data collected was filtered to ensure that the relevant data exclusively dedicated to pathogen protein interactions with proteins of the human cardiovascular system was retained. A UniProt accession number was used to identify the protein molecules uniformly that were collected from different sources. The pathogen names were also checked for differences in syntax/nomenclature and were transformed into the single uniform format on the basis of same UniProt Taxon identifier. Duplicate records were removed from the data to prevent redundancy.
3. *Data enrichment*
  The data was further processed and enriched with additional parameters. A list of unique host and pathogen proteins was extracted from the initial HPI data. Gene id numbers were added to this protein molecules list using db2db tool of the bioDBnet database. The unique protein entities of both human and pathogens were also enriched with information about their gene ontologies. The same species and different species interactors of host and pathogen proteins obtained from the UniProt database were added and also the drug target information obtained from DrugBank 3.0^39^. An indicator of the number of interactions (degree) was assigned to all the unique host proteins and pathogen proteins using MySQL command line. The Gene Ontology (GO) identifiers were also given a quantitative value on the basis of their occurrence in the finally processed data.
4. *Database development*
  After processing and gathering of final data, MySQL relational database management system (RDMS) was used to construct a database by making use of various MySQL tools. The data was uploaded on the MySQL server localhost using MySQL workbench and query commands were made in the Command line Client of MySQL server. Several constraints like primary keys and foreign keys were assigned to several entities of tables in the database to remove and prevent further redundancy and ambiguities from the data in order to make a normalized database. Next, we proceeded to deploy our database in the form of a website. For this purpose, we used asp.net tools (C#) to develop the backend of the website environment and to link the MySQL database. The front end of the website was developed using HTML, CSS, JQuery and JavaScript. The use of Microsoft Visual Studio was also made to connect the asp.net backend environment with the HTML script front end so as to develop the whole web application. The web interface includes several search options for specific query and retrieval of data pertaining to ‘Disease’, ‘Pathogen-Specific Interactions’, ‘Protein-Specific Interactions’, ‘Gene Ontologies’, ‘Interaction Detection Methods’ and ‘Interactors and Drug Targets’. The web interface also has a ‘Contact us’ page which includes all the documentation about the database and also a query form for the submission of any type queries or bug reports by the user.
5. *Data analysis*

Analysis of the database was done using R studio, MySQL and Microsoft Excel. R script was used to sort the entities and quantitatively measure the occurrence of pathogens, pathogen proteins, pathogen taxon categories, and host proteins, interactions per disease term, gene ontologies, detection methods, interaction types, gene ids and the number of interactors of host and pathogen proteins in the data. R script was also used to perform drug target analysis to look for the number of interactions of drug target proteins in the data and their gene ontology pattern. The graphs showing the frequency of the top ten maximally occurring entities of the database were made using Microsoft Excel. Quantitative analysis for the number of interactions of host and pathogen proteins was done in MySQL. A degree value was assigned to each unique protein (both human and pathogen) on the basis of the number of their interactions in the data. The high degree (highly interacting) proteins were obtained using a cutoff greater than three standard deviations from the mean.

## Supplementary information

Supplementary File Singh et al, 2019

## Data Availability

The authors confirm that the data supporting the findings of this study is available at http://morcvd.sblab-nsit.net/About.
