# Supplementary material for: MorCVD: A Unified Database for Host-Pathogen Protein-Protein Interactions of Cardiovascular Diseases Related to Microbes
Source: Sci Rep. 2019 Mar 11;9:4039. doi: 10.1038/s41598-019-40704-5 (PMC6411875; doi:10.1038/s41598-019-40704-5)
Supplement: Supplementary file 1 — Supplementary File Singh et al, 2019 [file 41598_2019_40704_MOESM1_ESM.pdf]

Supplementary File

Title: MorCVD: A Unified Database for Host-Pathogen Protein-Protein Interactions of Cardiovascular Diseases Related to Microbes.

Nirupma Singh[1], *Venugopal Bhatia*[1,+], *Shubham Singh*[2,+] *and Sonika Bhatnagar*[1,*]

[1]*Computational and Structural Biology Laboratory,*

Division of Biological Sciences and Engineering,

Netaji Subhas University of Technology,

Dwarka, New Delhi 110078

India.

[2]*Division of Computer Engineering*

Netaji Subhas University of Technology,

Dwarka, New Delhi 110078

India.

[+] These authors contributed equally to this work

***Corresponding author:**

Dr. Sonika Bhatnagar

Computational and Structural Biology Laboratory,

Division of Biological Sciences and Engineering,

Netaji Subhas University of Technology,

New Delhi 110078

India

Phone: +91-11-25099027, Fax: +91-11-25099022.

Email: ecc999@gmail.com; sbhatnagar@nsit.ac.in

Documentation

Introduction

Cardiovascular diseases are one of the world's leading global killers, accounting for almost 31% of all global deaths. The role microorganisms play in CVDs is controversial and not completely understood as of today which makes it an important avenue for research. MorCVD intends to service the needs of such kind of research since identification and analysis of host pathogen protein interactions is essential for analysis of infectious diseases and to devise pathway intervention strategies. The host pathogen protein-protein interaction data has been curated from various databases, along with various other relevant parameters to enhance the information imparted by the data to users.

Release Date: June 15, 2018

Citation:

Singh,N., Bhatia,V., Singh, S., Bhatnagar, S. MorCVD: A unified database for host-pathogen protein-protein interactions of cardiovascular diseases related to microbes. (2018) (Communicated).

How to use

The contents of the database have been arranged and presented keeping ease of use in mind. We describe here the contents of each page and the operating procedure to access relevant data.

Home/MorCVD: The home page provides a brief description of the database detailing the organisation and search options that are part of the database and the essential parameters listed across the various pages. The database contains the following search options:

1. **Disease**: The Disease page allows the user to select from a list of Cardiovascular diseases and then lists the following information:
    - The Name of the pathogen involved in the condition.
    - The Taxonomy id for the pathogen.
    - The pathogen protein involved in a certain pathway.
    - The host protein interacting with this pathogen protein.
    - The relevant PubMed ID listing the interaction.
    - The Gene Symbol for host protein.
    - The Gene Symbol for pathogen protein.
    - The Uniprot entry for host protein.
    - The Uniprot entry for pathogen protein.

- The Source database from where the interaction was derived.
- Confidence Score for the interaction

1a. Using the Search bar: Functionality for searching for specific host/pathogen proteins by id has been introduced by search within results bar which appears on all result pages of the disease tab. This enables the user to zero in on specific protein interaction records within the results, by entering the host or pathogen protein id thus enhancing data accessibility.

2. **Pathogen Specific Interactions**: The page displays a dropdown of 432 pathogens to select from and lists the following information:
   - The first column lists the pathogen proteins belonging to the pathogen selected by the user.
   - The second column lists the host proteins, the respective pathogen proteins interact with.
   - The third column lists the type of the respective interactions.

3. **Protein Specific Interactions**: The page lets us choose for proteins from either the host or the pathogen. The user needs to supply the Uniprot accession number for the required protein. Additionally, the user is required to select the pathogen name as well in case pathogen is selected. Once the user inputs the necessary data the page displays the following information:
   - In case Pathogen has been selected:
     1. The human host proteins that have an interaction with the given pathogen protein, present in the database.
     2. The disease in which particular interaction is involved.
     3. The respective Gene ID of human protein.
     4. The following three degrees listed:
        a. Degree 1: This degree states the no. of pathogen proteins; the host protein has interactions with, that have been listed in the database.
        b. Degree 2: This degree lists the number of microorganisms; whose proteins have an interaction with the given host protein, present in the database.
        c. Degree 3: This degree is the sum of Degree 1 and Degree 2.
   - In case Human has been selected:
     1. The pathogen proteins that have an interaction with the given human protein, present in the database.
     2. The disease in which particular interaction is involved.
     3. The respective Gene ID of pathogen protein.
     4. The following three degrees are listed:
        a. Degree 1: This degree states the number of host proteins, whose interactions are present in the database, that interact with the respective pathogen protein.

b. Degree 2: This degree lists the number of pathogens in which the given protein occurs, for the data present in the database.
c. Degree 3: This degree is the sum of Degree1 and Degree 2.

5. **Gene Ontologies**: This page requires the user to select between human or pathogen from the dropdown "Host Organism", the user then needs to supply the relevant Uniprot Accession number. The following data is then displayed:
   - The Gene Ontology Id for the protein.
   - The function of the protein, corresponding to the Gene Ontology Id.
   - The Degree: This is the frequency for the protein possessing the given function and GO Id for all interactions present in the database.

6. **Interaction Detection Method**: The page allows the user to list the interaction data that can be obtained by a particular Interaction Detection Method, selected from a drop-down menu. The page lists the host protein, pathogen protein and the interaction type for each reaction.
   There is also a section to list the data which is as follows:
   Multiple Methods: This enables the user to query records based on the number of interaction methods through which the HPIs may be detected. For instance, selecting 2 from the drop down menu would display all interactions that are detected using 2 interaction methods. The drop down menu gives the user to select from anywhere between a minimum of 2 and a maximum of 11 detection methods which is the maximum number of methods observed in a particular record.

   A brief description of the interaction detection methods can be found in Supplementary Table S1 at the end of this section.

7. **Interactors**: This page allows us to view all the interactors from the same as well as different species that may or may not be part of the interactions listed in the database, for a particular pathogen or host protein. Additionally, we also provide information regarding whether the given protein happens to be a drug target or not. The information is listed in the form of two sections:
   - The first section states with a "Yes" or "No" whether the protein given by the user is a drug target.
   - The second section lists the interactor proteins for the given protein. Providing such interactor proteins may be significant in case of investigation for cross talk in different pathways possessing a certain degree of synergism.

FAQs

- **What is MorCVD?**
  MorCVD is a database containing data of host - pathogen protein-protein interactions (HPPPIs) involved in cardiovascular diseases (CVDs). This database covers 19 major

conditions such as Viral myocarditis, Bacterial endocarditis and Dilated Cardiomyopathy, along with other microbial CVDs.

- **Why are HPIs important?**
  Identification and analysis of host pathogen protein interactions is essential for analysis of infectious diseases and to devise pathway intervention strategies.

- **What are Confidence Scores?**
  The presence of false positives in the protein-protein interaction data is inevitable. It is thus necessary to have a metric for gauging the accuracy or likelihood of an interaction being a biologically relevant true positive. To this end confidence scores are assigned to host pathogen interactions. These confidence scores can be calculated using a wide variety of statistical methods and help give weightage to the correctness of an interaction. The confidence scores have been taken from the various databases from where the interaction data was originally obtained. There is also the option to sort the records as per the confidence scores from high scores to low scores and vice versa.

  The calculation of the confidence scores themselves may be done using various methods. These scores may combine a variety of schemes such as: scoring based on the number of publications supporting the interaction, scores based on detection methods, scores based on interaction types, scores based on gene ontologies and so on. Alternatively, the different scores may be combined and normalised to probability values between 0 and 1 with 1 being the score reflecting the highest confidence value or the highest probability of an interaction being true.

- **What are Gene Ontology Ids?**
  The objective of Gene Ontology ids are to provide controlled vocabularies for the description of the biological process, molecular function, and cellular component of gene products.

- **What do the Degrees mean on the Protein Specific Interactions page?**
  For human proteins the following three degrees have been listed:

  Degree 1: This degree states the no. of pathogen proteins; the host protein has interactions with, that have been listed in the database.
  Degree 2: This degree lists the number of microorganisms; whose proteins have an interaction with the given host protein, present in the database.
  Degree 3: This degree is the sum of Degree 1 and Degree 2.

  For pathogen proteins the following three degrees have been listed:

  Degree 1: This degree states the number of host proteins, whose interactions are present in the database, that interact with the respective pathogen protein.

Degree 2: This degree lists the number of pathogens in which the given protein occurs, for the data present in the database.
Degree 3: This degree is the sum of Degree1 and Degree 2.

- **Where can I refer to the Uniprot accession proteins for entry in pages such as Gene Ontologies and Protein Specific Interactions?**
  There is no provision as such to explicitly list the human and the pathogen proteins present in the database separately, however one may refer to the pathogen specific interactions or disease page information and select a protein from the information that's listed for a query.

- **How do I contact the team?**
  Click on the contact us page where you may write to us your query. Alternatively information on the members of the team has been given here.

- **Where can we report bugs?**
  You may mention all bugs and errata in the form on the contact us page.

Contributing Databases:

[1] Ammari, M.G., et al., *HPIDB 2.0: a curated database for host–pathogen interactions.* Database: The Journal of Biological Databases and Curation, 2016. **2016**: p. baw103.

[2] Chatr-aryamontri, A., et al., *MINT: the Molecular INTeraction database.* Nucleic Acids Research, 2007. **35**(Database issue): p. D572-D574.

[3] Stark, C., et al., *BioGRID: a general repository for interaction datasets.* Nucleic Acids Research, 2006. **34**(Database issue): p. D535-D539.

[4] Launay, G., et al., *MatrixDB, the extracellular matrix interaction database: updated content, a new navigator and expanded functionalities.* Nucleic Acids Research, 2015. **43**(Database issue): p. D321-D327

[5] The UniProt Consortium, *UniProt: the universal protein knowledgebase.* Nucleic Acids Research, 2017. **45**(Database issue): p. D158-D169.

[6] Goll, J., et al., *MPIDB: the microbial protein interaction database.* Bioinformatics, 2008. **24**(15): p. 1743-1744.

[7] Navratil, V., et al., *VirHostNet: a knowledge base for the management and the analysis of proteome-wide virus–host interaction networks.* Nucleic Acids Research, 2009. **37**(Database issue): p. D661-D668.

[8] Lynn, D.J., et al., *InnateDB: facilitating systems-level analyses of the mammalian innate immune response.* Molecular Systems Biology, 2008. **4**: p. 218-218

[9] Xenarios, I., et al., *DIP: the Database of Interacting Proteins.* Nucleic Acids Research, 2000. **28**(1): p. 289-291

[10] Brown, K.R. and I. Jurisica, *Unequal evolutionary conservation of human protein interactions in interologous networks.* Genome Biology, 2007. **8**(5): p. R95-R95

[11] Kerrien, S., et al., *The IntAct molecular interaction database in 2012.* Nucleic Acids Research, 2012. **40**(Database issue): p. D841-D846.

Supplementary Table S1: A brief description of the Interaction detection methods in the database

1. **Acetylation reaction**: Acetylation reactions introduce an acetyl functional group into a chemical compound. These reactions can affect K,C,A,D,E,Q,G,I,K,M,P,S,T,Y,V residues, which is an indicator of protein protein interactions.
2. **Adp ribosylation reaction**: Experiment involves the use of anti-poly(ADP-ribose) antibody for detection of the ADP-ribose moiety attached with particular protein as an indicator of protein-protein interaction.
3. **Affinity chromatography:** An interaction is inferred when a bait protein is affinity captured from cell extracts by either polyclonal antibody or epitope tag and the associated interaction partner is identified by Western blot with a specific polyclonal antibody or second epitope tag, Northern blot, RT-PCR, affinity labelling, sequencing, mass spectroscopic methods or microarray analysis
4. **Affinity technology:** This class of approaches is characterised by the use of affinity resins as tools to purify molecule of interest (baits) and their binding partners. The baits can be captured by a variety of high affinity ligands linked to a resin - for example, antibodies specific for the bait itself, antibodies for specific tags engineered to be expressed as part of the bait or other high affinity binders such as glutathione resins for GST fusion proteins, metal resins for histidine-tagged proteins.
5. **Alanine scanning:** This approach is used to identify the residues that are involved in an interaction. Several variants of the native protein are prepared by sequentially mutating each residue of interest to an alanine. The mutated proteins are expressed and probed in the binding assay.
6. **Anti-bait coimmunoprecipitation:** A specific antibody for the molecule of interest (bait) is available, this is used to generate a high affinity resin to capture the endogenous bait present in a sample.
7. **Antibody array**: This assay involves use of a membrane on which 60 antibodies against different cell cycle proteins are pre-immobilized in a pre-determined position. This antibody-immobilized membrane was allowed to incubate with the whole cell lysates. The protein complexes were captured on the membrane, and then detected by immunoblotting using anti-c-Myc antibody
8. **Anti-tag coimmunoprecipitation:** A specific antibody for the molecule of interest is not available, therefore the bait protein is expressed as a hybrid protein fused to a tag peptide/protein for which efficient and specific antibodies or a specific ligand are available.
9. **Bimolecular fluorescence complementation (BiFC):** BiFC is an assay for determination of protein interactions and/or their location in living cells. This approach is based on complementation between two non- fluorescent fragments of a protein fluorophore such as green fluorescent protein (GFP) or its derivatives. Interactions between proteins fused to each fragment bring the fragments together resulting in the reconstitution of a fully functional flourophore that can be identified through fluorescence spectroscopy or microscopy.
10. **Biochemical:** Referring to definition on BioGRID, an interaction is inferred from the biochemical effect of one protein upon another *in vitro*, for example, GTP-GDP exchange activity or phosphorylation of a substrate by a kinase. The “bait” protein executes the activity on the substrate “hit” protein. A Modification value is recorded for interactions of this type with the possible values Phosphorylation, Ubiquitination, Sumoylation, Dephosphorylation, Methylation, Prenylation, Acetylation, Deubiquitination, Proteolytic Processing, Glucosylation, Nedd(Rub1)ylation, Deacetylation, No Modification, Demethylation.
11. **BioID:** BioID is a unique method to screen for physiologically relevant protein interactions that occur in living cells. This technique harnesses a promiscuous biotin ligase to biotinylate proteins based on proximity. The ligase is fused to a protein of interest and expressed in cells, where it biotinylates proximal endogenous proteins. Because it is a rare protein modification in nature, biotinylation of these endogenous proteins by BioID fusion proteins enables their selective isolation and identification with standard biotin-affinity capture.
12. **Bioluminescence resonance energy transfer**: In this variation of the FRET assay the donor fluorophore is replaced by a luciferase (typically Renilla luciferase). In the presence of its substrate, the luciferase catalyses a bioluminescent reaction that excites the acceptor fluorophore through a resonance energy transfer mechanism. As with FRET the energy transfer occurs only if the protein fused to the luciferase and the one fused to the acceptor fluorophore are in close proximity (10-100 Å).

13. **Blue native page:** Blue native page can be used to determine native protein masses and oligomeric states and to identify physiological protein-protein interactions. Native complexes are recovered from gels by electroelution or diffusion and are used for 2D crystallization and electron microscopy or analyzed by in-gel activity assays or by native electroblotting and immunodetection.
14. **Chromatin immunoprecipitation assay:** Chromatin immunoprecipitation (ChIP) is a powerful approach that allows one to define the interaction of factors with specific chromosomal sites in living cells. An antibody against a protein suspected of binding a given cis-element is used to immunoprecipitate fragmented chromatin fragments. Cells or tissue may first be briefly treated with an agent such formaldehyde to crosslink proteins to DNA. Nucleic acids are then identified by sequencing, for example polymerase chain reaction analysis of the immunoprecipitate with primers flanking the cis-element or next-generation sequencing techniques
15. **Chromatography technology:** Used to separate and/or analyse complex mixtures. The components to be separated are distributed between two phases: a stationary phase (bed) and a mobile phase which percolates through the stationary bed. The nature of the two phases determines the separation criteria exploited by the column such as affinity, ionic charges, size or hydrophobicity of the molecules under analysis. Each type of column can be implemented with the mobile phase under atmospheric or high pressure condition.
16. **Circular dichroism (CD):** CD spectroscopy is a useful technique for studying protein-protein interactions in solution. CD in the far ultraviolet region (178-260 nm) arises from the amides of the protein backbone and is sensitive to the conformation of the protein. Thus, CD can determine whether there are changes in the conformation of proteins when they interact.
17. **Classical fluorescence spectroscopy:** Proteins contain endogenous fluorophores such as tryptophan residue and heme or flavins groups. Protein folding and protein-protein interaction can be studied by monitoring changes in the tryptophan environment detected by changes in its intrinsic fluorescence. Changes in the fluorescence emission spectrum on complex formation can occur either due to a shift in the wavelength of maximum fluorescence emission or by a shift in fluorescence intensity caused by the mixing of two proteins. The interaction of two proteins causes a shift in the fluorescence emission spectrum relative to the sum of the individual fluorescence spectra, resulting in a difference spectrum [F (complex)-2 F (sum)], which is a measurable effect of the interaction. Loss of fluorescence signal from a substrate can be used to measure protein cleavage.
18. **Cleavage assay:** Experiments involve the cleavage of a biomolecule either into its component parts or sub-parts.
19. **Coimmunoprecipitation (Co-IP):** Co-IP works by selecting an antibody that targets a known protein that is believed to be a member of a larger complex of proteins. By targeting this known member with an antibody it may become possible to pull the entire protein complex out of solution and thereby identify unknown members of the complex.
20. **Co-localization by fluorescent probes cloning:** Co-localization of fluorescent probes is commonly used in cell biology to discern the proximity of two proteins in the cell.
21. **Colocalization by fluorescent probes cloning:** The technique involves observation of spatial overlap between fluorescent labels each with a distinct emission wavelength to observe whether proteins in question are spatially near to one another
22. **Co-localization by immunostaining:** The subcellular location of a protein can be demonstrated by treating cells fixed on a microscope slide with an antibody specific for the protein of interest. A secondary antibody conjugated with a reactive enzyme (e.g. horseradish peroxidase) is then added. Following a washing step to remove the unbound secondary ligand, a chromogenic substrate (e.g. 3,3', 5,5' tetramethyl benzidine chromogen [TMB]) is converted to a soluble coloured product by the conjugated enzyme and can then be visualised by standard microscopic techniques.
23. **Colocalization by immunostaining:** The subcellular location of a protein can be demonstrated by treating cells fixed on a microscope slide with an antibody specific for the protein of interest. A secondary antibody conjugated with a reactive enzyme is then added. Following a washing step to remove the unbound secondary ligand, a chromogenic substrate is converted to a soluble coloured product by the conjugated enzyme and can then be visualised by standard microscopic techniques.
24. **Colocalization:** An interaction is inferred from two proteins that co-localize in the cell by indirect

	immunofluorescence only when in addition, if one gene is deleted, the other protein becomes mis-localized. This also includes co-dependent association of proteins with promoter DNA in chromatin immunoprecipitation experiments and in situ proximity ligation assays
25.	**Comigration in gel electrophoresis:** Determination of interaction between two molecules is done by their very close proximity or the overlap of their relative bands in a gel.
26.	**Co-migration in gel electrophoresis:** The interaction of two molecules is determine by their very close proximity or the overlap of their relative bands in a gel.
27.	**Comigration in non-denaturing gel electrophoresis** : The interaction of two molecules is determine by their very close proximity or the overlap of their relative bands in a gel.
28.	**Co-migration in SDS page:** Method allowing the detection of strong interactions between two molecules by their very close proximity or the overlap of their relative bands in a denaturing SDS gel.
29.	**Comigration in SDS page:** This Method allowing the detection of strong interactions between two molecules by their very close proximity or the overlap of their relative bands in a denaturing SDS gel.
30.	**Competition binding:** Competitive binding experiments measure equilibrium binding of a single concentration of ligand at various concentrations of an unlabelled competitor. Analysis of these data gives the affinity of the receptor for the competitor.
31.	**Confocal microscopy:** A confocal is a standard epifluorescence microscope with improvement essentially coming from the rejection of out-of-focus light interference. Confocal imaging system achieves this by two strategies: a) by illuminating a single point of the specimen at any one time with a focused beam, so that illumination intensity drops off rapidly and b) by the use of blocking a pinhole aperture in a conjugate focal plane to the specimen so that light emitted away from the point in the specimen being illuminated is blocked from reaching the detector. Only the light from the single point illuminated of the specimen passing through the image pinhole is detected by a photodetector. Usually a computer is used to control the sequential scanning of the sample and to assemble the image for display onto a video screen.
32.	**Copurification:** An interaction is inferred from the identification of two or more protein subunits in a purified protein complex, as obtained by several classical biochemical fractionation steps, or else by affinity purification and one or more additional fractionation steps
33.	**Co-sedimentation in solution:** The ultracentrifuge can be used to characterise and/or purify macromolecules in solution according to their mass and hydrodynamic properties. Sedimentation studies provide information about the molecular weight and shape of a molecule. It is also possible to measure the association state of the sample. Both the mass of a molecule and its shape, that influences the friction forces and diffusion that counterbalances gravity, determine the sedimentation speed.
34.	**Co-sedimentation through density gradient:** Sedimentation through a density gradient measures the sedimentation rate of a mixture of proteins through either a glycerol or sucrose gradient. Two interacting proteins will sediment mostly as a complex at concentrations above the binding constant. By varying the concentration of one or both of the complex constituents and taking into account the dilution of the species during sedimentation, one can reasonably accurately estimate the binding constant.
35.	**Cosedimentation using density gradient**: Sedimentation through a density gradient measures the sedimentation rate of a mixture of proteins through either a glycerol or sucrose gradient. Two interacting proteins will sediment mostly as a complex at concentrations above the binding constant. By varying the concentration of one or both of the complex constituents and taking into account the dilution of the species during sedimentation, one can reasonably accurately estimate the binding constant.
36.	**Co-sedimentation:** Separation of a mixture of molecules under the influence of a force such as artificial gravity. Molecules sedimenting together are assumed to interact.
37.	**Cross-linking study:** Analysis of complexes obtained by input of energy or chemical treatments, or by introducing cysteines followed by oxidation to promote the formation of covalent bonds among molecules in close proximity.

38. **Cytoplasmic complementation assay:** Protein complementation assay is performed by dissecting a cytoplasmic protein activity and restoring it through the two hybrid proteins interaction.
39. **Dephosphorylation assay**: Under phosphatase assay, Experiments involve measuring the catalysis of the reaction: a phosphosubstrate + H2O= a substrate + phosphate.
40. **Dynamic light scattering:** In dynamic light scattering, particle diffusion in solution gives rise to fluctuations in the intensity of the scattered light on the microsecond scale. The hydrodynamic radius of the particles can be easily calculated.
41. **Electron Microscopy**: Electron microscopy uses a beam of electrons to illuminate a sample and achieve much higher spatial resolution than light microscopy. Transmission electron microscopy generates an image of the internal structure of a thin sample. Scanning electron microscopy generates a topological image of a sample.
42. **Electron tomography:** Electron tomography is a technique for obtaining detailed 3D structures of sub-cellular macro-molecular objects.
43. **Electrophoretic mobility shift assay:** This method proves the interaction between a nucleic acid and a protein partner. On the same electrophoresis gel 1 lane is loaded with a nucleic acid of known sequence, a second lane is loaded with the same nucleic acid together with a purified protein (or a protein mixture). The nucleic acid is often radio-labelled to enable visualisation by autoradiography. Comparison of the nucleic acid migration in the two lanes enables the retardation of the nucleic acid due to its interaction with a protein to be observed.
44. **Enzyme linked immunosorbent assay:** Following non-covalent binding of a purified primary ligand to a solid phase, a blocking reagent is added to prevent any non-specific binding. A specific antigen is then allowed to bind to the primary ligand. Unbound antigen is removed by washing and a secondary antibody conjugated to an enzyme (e.g. horseradish peroxidase) is added. Following a washing step to remove unbound secondary ligand, the extent to which a chromogenic substrate (e.g. 3,3', 5,5' tetramethyl benzidine chromogen [TMB]) is converted to a soluble coloured product by the conjugated enzyme in a given time is determined by spectrophotometry using a standard microplate absorbance reader.
45. **Experimental interaction detection**: Inhibition of enzymatic activity of one protein by another was taken as an indicator of protein-protein interactions.
46. **Far western blotting:** Proteins are fractionated by PAGE (SDS-polyacrylamide gel electrophoresis), transferred to a nitrocellulose membrane and tested for the ability to bind to a protein, a peptide, or any other ligand. Cell lysates can also be fractionated before gel electrophoresis to increase the sensitivity of the method for detecting interactions with rare proteins. Denaturants are removed during the blotting procedure, which allows many proteins to recover (or partially recover) activity. However, if biological activity is not recoverable, the proteins can be fractionated by a non-denaturing gel system. This variation of the method eliminates the problem of activity regeneration and allows the detection of binding when the presence of a protein complex is required for binding. The protein probe can be prepared by any one of several procedures, while fusion affinity tags greatly facilitate purification. Synthesis in E. coli with a GST fusion, epitope tag, or other affinity tag is most commonly used. The protein of interest can then be radioactively labelled, biotinylated, or used in the blotting procedure as an unlabelled probe that is detected by a specific antibody.
47. **Field flow fractionation:** A separation technique where a field is applied to a mixture perpendicular to the mixtures flow. The filed can be gravitational, centrifugal, magnetic or a cross flow of fluids.
48. **Filamentous phage display:** Filamentous phages (M13, f1, fd) have been extensively used to develop and implement the technology of phage display. Repertoires of relatively short peptides of random amino acid sequences or cDNA libraries have been constructed and searched successfully. Most experiments have taken advantage of the ability to assemble phages decorated with hybrid versions of the receptor protein pIII or of the major coat protein pVIII. Both systems allow the display of foreign peptides by fusion to the amino-terminus of the capsid protein but differ in the number of peptide copies that can be displayed on each phage particle. Display libraries of very diverse protein fragments have been constructed by fusing either genomic or cDNA fragments to gene III or gene VIII.
49. **Filter binding**: Experiments involving the binding of proteins to nitrocellulose filters after SDS PAGE. The binding of proteins was taken as an indicator of protein protein interactions.

50.	**Fluorescence microscopy:** Fluorescence microscopy uses fluorescence and phosphorescence instead of, or in addition to, scattering, reflection, and attenuation or absorption, to study properties of organic or inorganic substances.
51.	**Fluorescence polarization spectroscopy:** Because of the long lifetimes of excited fluorescent molecules (nanoseconds), fluorescence can be used to monitor the rotational motion of molecules, which occurs on this timescale. This is accomplished experimentally by excitation with plane-polarized light, followed by measurement of the emission at parallel and perpendicular planes. Since rotational correlation times depend on the size of the molecule, this method can be used to measure the binding of two proteins because the observed polarization increase when a larger complex is formed. A fluorescence anisotropy experiment is normally carried out with a protein bearing a covalently added fluorescent group, which increases both the observed fluorescence lifetime of the excited state and the intensity of the fluorescent signal. Residue modification can be assessed by addition of an antibody which binds to the modified residue and alters the molecular weight of the complex. A variation of this technique has been used to show interaction of a DNA binding protein with another protein. In this case the DNA rather than protein is fluorescently labelled.
52.	**Fluorescence recovery after photobleaching(FRAP)**: Fluorescence recovery after photobleaching is based on irreversibly bleaching a pool of fluorescent probes and monitoring the recovery in fluorescence due to movement of surrounding intact probes into the bleached spot
53.	**Fluorescence-activated cell sorting:** Cells in suspension flow through a laser beam, the scattered light or emitted fluorescence is measured, filtered and converted to digital values. Cells can be sorted according to their properties. Using flow cytometry, any fluorescent or light scattering experiment can be carried out on entire cells. With this instrument, interactions occurring either on cell surfaces or in any other sub cellular location can be studied by using suitable fluorescent labels.
54.	**Fluorescent resonance energy transfer (FRET):** FRET is a quantum mechanical process involving the radiationless transfer of energy from a donor fluorophore to an appropriately positioned acceptor fluorophore. The fluorophores are genetically fused to the protein in analysis and cotransfected. Three basic conditions must be fulfilled for FRET to occur between a donor molecule and acceptor molecule. First, the donor emission spectrum must significantly overlap the absorption spectrum of the acceptor. Second, the distance between the donor and acceptor fluorophores must fall within the range 20 to 100 Angstrom. Third, the donor and acceptor fluorophores must be in favourable orientations.
55.	**Footprinting**: DNase I footprinting assay is an *in vitro* method to identify the specific site of DNA binding proteins. It not only finds the target protein that binds to specific DNA, but also identifies which sequence the target protein is bound. This technique can be used to study interactions between proteins and DNA both outside and within cells
56.	**gal4-vp16 complementation:** A chimeric protein consisting of the GAL4 DNA-binding domain (aa 1-147 of GAL4) and a transcriptional activation domain from the herpes simplex virus protein VP16 (either aa 411-490 or aa 411-455) can specifically activate transcription of a reporter gene located downstream ofGAL4 DNA binding sites and the E1B minimal promoter. Similarly, two chimeric proteins, one encoding a chimeric GAL4 protein and the other encoding a chimeric VP16 protein, can activate the reporter gene, if the domains fused to the GAL4 and VP16 sequences can complex with appropriate conformation. However, if the domains fused to the GAL4 and VP16 sequences do not interact specifically to form a + complex that reconstitutes GAL4 function, the reporter gene cannot be activated.
57.	**GDP/GTP exchange assay:** Experiments monitoring interactions of GTP-GDP exchange factors.
58.	**Genetic interference:** This term refers to methods that aim at interfering with the activity of a specific gene by altering the gene regulatory or coding sequences. This goal can be achieved either by a classical genetic approach (random mutagenesis followed by phenotype characterization and genetic mapping) or by a reverse genetics approach where a gene of interest is modified by directed mutagenesis.
59.	**Glycosylase assay**: Glycosylation of proteins leading to inhibition of protein synthesis and death of target cells was taken as indicator of protein protein interactions.
60.	**GST pull down:** The bait protein is expressed and purified as a fusion to the glutathione S-transferase protein. The bait protein is normally attached to a glutathione sepharose resin or alternatively to a

	support containing an anti-GST antibody.
61.	**Gtpase assay**: Experiment to measure the catalysis of the reaction: GTP + H2O = GDP + phosphate.
62.	**His pull down:** The pull-down assay is a method used to determine a physical interaction between two or more proteins. In a pull-down assay, a bait protein is tagged with His tags and captured on an immobilized affinity ligand specific for the tag, thereby generating a secondary affinity support for purifying other proteins that interact with the bait protein.
63.	**Imaging technique:** Methods that provide images of molecules at various resolutions depending on the technology used.
64.	**In vitro**: Refers to analysis of the PPI in vitro by transformation of the fusion protein in E.Coli.
65.	**In-gel kinase assay:** Substrate protein radio-labelled by kinase transferring an isotope of phosphate from the nucleotide. Substrate isolated by gel electrophoresis and radio-labelling confirmed by autoradiography.
66.	**Interactome parallel affinity capture:** A specific pull down method where the protein of interest (bait) is endogenously expressed with at least two affinity tags (GFP, FLAG or others). The bait is purified in parallel using different purification protocols in contrast to tandem affinity purification (TAP)
67.	**Ion-exchange chromatography:** Stable complexes and their component proteins can be separated on the basis of their net charge by ion-exchange chromatography. If a protein has a net positive charge at pH 7, it will usually bind to a column of beads containing carboxylate groups, and can then be eluted by increasing the concentration of sodium chloride or another salt in the eluting buffer by competition of sodium ions with positively charged groups on the protein for binding to the column. Protein that have a low density of net positive charge will tend to emerge first, followed by those having a higher charge density. Positively charged complexes or proteins (cationic proteins) can be separated on negatively charged carboxymethyl-cellulose (CM-cellulose) columns. Conversely, negatively charged complexes or proteins (anionic proteins) can be separated by chromatography on positively charged diethylaminoethyl-cellulose (DEAE-cellulose) columns.
68.	**Isothermal titration calorimetry:** Isothermal titration calorimetry (ITC) measures directly the energy associated with a chemical reaction triggered by the mixing of two components.
69.	**Lexa b52 complementation:** Yeast two-hybrid system using Escherichia coli LexA amino acids 1-202 as the DNA-binding domain (BD), E. coli B42 acidic sequence as the activation domain (AD), and two reporters, lacZ and LEU2, each containing upstream LexA binding elements.
70.	**Light scattering:** Experiments may involve Dynamic Light Scattering (DLS) or Static Light scattering for protein protein interactions. DLS allows allows particle sizing down to 1 nm diameter. The sample is illuminated by a laser beam and the fluctuations of the scattered light are detected at a known scattering angle θ by a fast photon detector.
71.	**Lipase assay:** Lipase activity was determined by hydrolysis of the substrate pNPP and subsequent release of p-nitrophenol, which was detected by measuring the absorbance of the reaction solution at 405 nm
72.	**Luminescence based mammalian interactome mapping:** This strategy uses a luciferase enzyme fused to proteins of interest, which are then coexpressed with individual epitope-tagged partners in mammalian cells. Their interactions are determined by performing an enzymatic assay on anti-tag immunoprecipitates.
73.	**Mammalian protein-protein interaction trap:** The MAPPIT (mammalian protein-protein interaction Trap) is a screening method for protein-protein interaction in mammalian cells, based on the reconstitution of a membrane STAT (signal transducers and activators of transcription) receptor. The bait protein is fused to a STAT recruitment-deficient receptor and the prey protein to a functional STAT recruitment sites. In such a configuration, a given bait prey interaction restores a STAT-dependent responses leading to the expression of a reporter gene. This system, enable to demonstrate not only protein interaction but also modification-independent and tyrosine phosphorylation- dependent interactions.
74.	**Mass spectrometry study of hydrogen/deuterium exchange:** Hydrogen-deuterium exchange (HDX) mass spectrometry (MS) can provide valuable information about binding, allostery, and other

	conformational effects of interaction in protein complexes. For protein-ligand complexes, where the ligand may be a small molecule, peptide, nucleotide, or another protein(s), a typical experiment measures HDX in the protein alone and then compares that with HDX for the protein when part of the complex.
75.	**Mass spectroscopy studies of complexes:** Mass spectrometric approaches to the study of macromolecular complexes permits the identification of subunit stoichiometry and transient associations. By preserving complexes intact in the mass spectrometer, mass measurement can be used for monitoring changes in different experimental conditions, or to investigate how variations of collision energy affect their dissociation.
76.	**Methyltransferase assay**: Modification of protein domains by methyltransferase was taken as an indicator of protein protein interactions.
77.	**Microscale thermophoresis:** Measurement of the directed movement of particles in a microscopic temperature gradient. Any change of the hydration shell of biomolecules due to changes in their structure/conformation results in a relative change of movement along the temperature gradient and is used to determine binding affinities, binding kinetics and activity kinetics. Events such as the phosphorylation of a protein or the binding of small molecules to a target can be monitored.
78.	**Molecular interaction**: Refers to employment of variety of techniques such as immunofluorescence, subcellular localization, fluorescence resonance energy transfer (FRET) assays and Yeast two-hybrid assays.
79.	**Molecular sieving:** In sizing columns (gel filtration), the elution position of a protein or of a complex depends on its Stokes radius. Molecules with a radius that is smaller than the bead size are retained and retarded by the interaction with the matrix. The observation that two proteins, loaded on a sieving column, elute in a fraction(s) corresponding to a MW that is larger than the MW of either protein may be taken as an indication that the two proteins interact. Further, this technique provides a conceptually simple method for evaluating the affinity of the interaction.
80.	**Myc tag coimmunoprecipitation:** Co-immunoprecipitation (co-IP) is a popular technique to identify physiologically relevant protein-protein interactions by using target protein-specific antibodies to indirectly capture proteins that are bound to a specific target protein. These protein complexes can then be analyzed to identify new binding partners, binding affinities, the kinetics of binding and the function of the target protein. Myc tags used here for bait proteins.
81.	**Nuclear magnetic resonance:** Nuclear Magnetic Resonance (NMR) is a type of spectroscopy whereby nuclei in a magnetic field absorb and re-emit electromagnetic radiation. It has many applications throughout the physical sciences and industry and is commonly used to study the structure of organic compounds
82.	**Peptide array:** Peptide arrays are powerful tools for the investigation of protein-protein and drug-protein interactions. For peptide-based drug design, with peptide microarrays it is possible to screen a high number of peptides on a small chip.
83.	**Peptide mass fingerprinting:** This approach leads to protein identification by matching peptide masses, as measured by mass spectrometry, to the ones calculated from in silico fragmentation of a protein sequence database.
84.	**Phage display:** Peptide sequences or entire proteins can be displayed on phage capsids by fusion to coat proteins to generate a library of fusion phages each displaying a different peptide. Such a library can then be exploited to identify specific phages that display peptides that bind to any given bait molecule for instance an antibody. The selection is performed by a series of cycles of affinity purification known as panning. The bait protein, immobilized on a solid support (plastic, agarose, sepharose, magnetic beads and others) is soaked in the phage mixture and that phage that remains attached to the bait is amplified and carried through a further affinity purification step. Each cycle results in an approximately 1,000-fold enrichment of specific phage and after a few selection rounds. DNA sequencing of the tight-binding phage reveals only a small number of sequences. Phage display panning experiments can be carried out either on libraries of peptides of random amino acid sequence or on libraries of displaying natural peptides obtained by inserting cDNA fragments into the phage vector (cDNA libraries). Libraries have been assembled on several different phages (Fd, Lambda or T7).

85.	**Phosphatase assay:** Experiments involve measuring the catalysis of the reaction: a phosphosubstrate + H2O= a substrate + phosphate.
86.	**Protease assay:** Measures the enzymatic hydrolysis of a peptide bond within a peptide or protein substrate.
87.	**Protein array:** The protein array technology allows the screening of biochemical activities or binding abilities of hundreds or thousands of protein samples in parallel. After synthesis and purification by high-throughput methodologies, the proteins are printed onto the chip by using an instrument (micro-arrayer) that is capable of spotting liquid samples in a reproducible manner onto a planar support. The ordered protein array can then be probed with labelled molecules to identify proteins that bind to the bait.
88.	**Protein complementation assay:** In the protein-fragment complementation assay, the proteins of interest ("Bait" and "Prey") are covalently linked at the genetic level to incomplete fragments of a third protein (known as the "reporter") and are expressed in vivo, Interaction between the "bait" and the "prey" proteins brings the fragments of the "reporter" protein in close enough proximity to allow them to reform and become the functional reporter protein. Typically enzymes which confer resistance to antibiotics, such as Dihydrofolate reductase or Beta-lactamase, or proteins that give colorimetric or fluorescent signals are used. The Bait protein is generally the protein under study and the methods are readily adaptable to high throughput mode.
89.	**Protein *in situ* array:** *In situ* or on-chip protein array methods use cell free expression systems to produce proteins directly onto an immobilising surface from co-distributed or pre-arrayed DNA or RNA, enabling protein arrays to be created on demand
90.	**Protein kinase assay:** The experiment measures the catalysis of the transfer of a phosphate group usually from ATP to a protein substrate.
91.	**Protein phosphatase assay**: The Phosphatase Assay kit is designed to measure the activity of phosphatases in biological samples and to screen for agonists and inhibitors of phosphatases.The Phosphatase Assay kit uses para-nitrophenyl phosphate (pNPP), a chromogenic substrate for most phosphatases, including alkaline phosphatases, acid phosphatases, protein tyrosine phosphatases and serine/threonine phosphatases.The phosphatases remove the phosphate group to generate p-nitrophenol, which is deprotonated under alkaline conditions to produce p-nitrophenolate that has strong absorption at 405nm wavelength.
92.	**Protein three hybrid:** Two hybrid assay performed with a third protein component co-transfected into a recombinant yeast strain together with a bait and a prey construct. Negative control shows that the interaction between the bait and the prey do not occur when the third protein is not co-transfected.
93.	**Proximity ligation assay:** Upon binding of two proximity probes (usually antibodies conjugated to DNA) to the same target protein complex, the oligonucleotides on the proximity probes are brought close together. These antibody-conjugated oligonucleotides hybridize to two connector oligonucleotides that are ligated to form a circular DNA molecule. This newly formed DNA-molecule can be amplified by rolling circle amplification. The resulting single-stranded DNA-molecule collapses into a bundle, which is detectable through hybridization of fluorescently labelled complementary oligonucleotides.
94.	**Proximity-dependent biotin identification:** A promiscuous biotin protein ligase is fused to the bait protein, neighbouring prey are then biotinylated. The biotin tag may be used for isolation and/or identification.
95.	**Pull down:** The pull-down assay is a method used to determine a physical interaction between two or more proteins. In a pull-down assay, a bait protein is tagged and captured on an immobilized affinity ligand specific for the tag, thereby generating a secondary affinity support for purifying other proteins that interact with the bait protein.
96.	**Small angle neutron scattering (SANS):** During a SANS experiment a beam of neutrons is directed at a sample. The neutrons are elastically scattered by a sample and the resulting scattering pattern is analyzed to provide information about the size, shape and orientation of some component of the sample.

97. **Solid phase assay:** Assay where at least one molecule under analysis is bound to a solid surface, such as a microplate wall or the sides of a tube, the other reactants being free in solution.
98. **Split firefly luciferase complementation:** Two proteins of interest, a bait and prey, which are genetically fused to amino- and carboxy-terminal fragments of firefly (Photinus pyralis) luciferase, are transiently expressed. Physical interactions of these bait and prey proteins reconstitute some of the luciferase activity and result in light emission in the presence of the luciferase substrate.
99. **Split luciferase complementation:** Two proteins of interest, a bait and prey, which are genetically fused to amino- and carboxy-terminal fragments of luciferase, are transiently expressed. Physical interactions of these bait and prey proteins reconstitute some of the luciferase activity and result in light emission in the presence of the luciferase substrate.
100. **Split luciferase complementation:** Two proteins of interest, a bait and prey, which are genetically fused to amino- and carboxy-terminal fragments of luciferase, are transiently expressed. Physical interactions of these bait and prey proteins reconstitute some of the luciferase activity and result in light emission in the presence of the luciferase substrate.
101. **Static light scattering:** In static light scattering, the average intensity of scattered light at multiple angles is measured. The data yield information on particle molecular weight, particle size and shape, and particle-particle interactions.
102. **Sumoylation assay:** SUMO stands for Small Ubiquitin-related Modifier. Covalent tagging of proteins is done by SUMO and SUMOylated proteins are recognized by ubiquitin and SUMO recognition motifs present on interacting proteins
103. **Surface Plasmon resonance:** This method measures formation of complex by monitoring changes in the resonance angle of light impinging on a gold surface as a result of changes in the refractive index of the surface.
104. **Tandem affinity purification:** TAP involves incorporating an epitope tag onto your protein of interest and performing a two-stage purification protocol. Tandem affinity purification (TAP) allows for rapid and efficient purification of epitope-tagged protein complexes from crude extracts under native conditions.
105. **Tap tag coimmunoprecipitation**: TAP involves incorporating an epitope tag onto your protein of interest and performing a two-stage purification protocol. Tandem affinity purification (TAP) allows for rapid and efficient purification of epitope-tagged protein complexes from crude extracts under native conditions.
106. **Two hybrid array:** Two-hybrid screening can be done in a colony array format, in which each colony expresses a defined pair of proteins. Because the particular protein pair expressed in each colony is defined by its position in the array, positive signals identify interacting proteins without further characterization, thus obviating the need for DNA purification and sequencing. The interrogation of a two-hybrid colony array usually involves a mating strategy in which every DNA binding domain hybrid (the bait) is tested against all activation domain hybrids (the preys) in a grid pattern. Arrays usually use full-length open reading frames.
107. **Two hybrid:** Two-hybrid screening can be done in a colony array format, in which each colony expresses a defined pair of proteins. Because the particular protein pair expressed in each colony is defined by its position in the array, positive signals identify interacting proteins without further characterization, thus obviating the need for DNA purification and sequencing. The interrogation of a two-hybrid colony array usually involves a mating strategy in which every DNA binding domain hybrid (the bait) is tested against all activation domain hybrids (the preys) in a grid pattern. Arrays usually use full-length open reading frames.
108. **Two prey hybrid pooling approach:** In the two hybrid pooling strategy sets of either both bait and prey hybrid vectors are mated or, more commonly, individual baits are mated against pools of prey. This approach required cloning baits and preys into both two-hybrid vectors, followed by pooling sets of the transformants. The positive double hybrid clones are the interacting partners. The pooling of both baits and prey molecules is now a rarely used technique as the pooling of baits often leads to misleading results.
109. **Two-hybrid pooling approach:** In the two-hybrid pooling strategy sets of either both bait and prey

hybrid vectors are mated or, more commonly, individual baits are mated against pools of prey. This approach required cloning baits and preys into both two-hybrid vectors, followed by pooling sets of the transformants. The positive double hybrid clones constitute the interacting partners. The pooling of both baits and prey molecules is now a rarely used technique as the pooling of baits often leads to misleading results.

110. **Validated two hybrid:** An interaction is inferred when a bait protein is expressed as a DNA binding domain (DBD) fusion, a prey protein is expressed as a transcriptional activation domain (TAD) fusion and the interaction is measured by reporter gene activation.

111. **Western blot:** Western blot is a procedure to identify and quantify proteins. A mixture of protein is first submitted to an electrophoresis in denaturing condition and then electro-transferred from the gel to a membrane. The membrane is then incubated with a primary antibody specific for a given protein or a specific residue modification in the sample under analysis. A secondary antibody, radiolabelled or fused to fluorophore or to a chromogenic enzyme, targets the first antibody and allows the visualisation of the protein band on the membrane.

112. **X ray scattering:** Small angle X-ray scattering (SAXS) is a high resolution characterization technique able to resolve features in the range between 1 and 100 nm. An important benefit of using this technique is that biological specimens can potentially be studied in their natural environment and high level of characterization of protein-protein interactions may be done.

113. **X-ray crystallography:** Analysis of a diffraction pattern generated by x-rays of a single crystal. X-ray crystallography enables visualization of protein structures at the atomic level and enhances the understanding of protein interaction and function

114. **Yeast display:** The proteins are displayed on the surface of the yeast *Saccharomyces cerevisiae* by fusion to signal sequences for protein secretion. This method is limited by the low efficiency of the yeast display system but can take full advantage of exploiting cell sorting methods (FACS) to isolate cells that display molecules with desired binding properties.

115. **β galactosidase complementation:** Beta-galactosidase activity can be used to monitor the interaction of chimeric proteins. Pairs of inactive beta gal deletion mutants are capable of complementing to restore activity when fused to interacting protein partners. Critical to the success of this system is the choice of two poorly complementing mutant moieties, since strongly complementing mutants spontaneously assemble and produce functional beta-gal activity detectable in absence of any fused protein fragment.

116. **β lactamase complementation:** This strategy is based on a protein fragment complementation assay (PCA) of the enzyme TEM-1 beta-lactamase. The approach includes a simple colorimetric in vitro assays using the cephalosporin nitrocefin and assays in intact cells using the fluorescent substrate CCF2/AM. The combination of in vitro colorimetric and in vivo fluorescence assays of beta-lactamase in mammalian cells permits a variety of sensitive and high-throughput large-scale applications.
